# Holmium laser enucleation of the prostate (HoLEP) is safe and effective in patients with high comorbidity burden

**DOI:** 10.1590/S1677-5538.IBJU.2022.0174

**Published:** 2023-02-18

**Authors:** Fabrizio Di Maida, Antonio Andrea Grosso, Riccardo Tellini, Samuele Nardoni, Sofia Giudici, Anna Cadenar, Vincenzo Salamone, Luca Lambertini, Matteo Salvi, Andrea Minervini, Agostino Tuccio

**Affiliations:** 1 Department of Experimental and Clinical Medicine University of Florence Florence Italy Department of Experimental and Clinical Medicine, University of Florence - Unit of Oncologic Minimally Invasive Urology and Andrology, Careggi Hospital, Florence, Italy

**Keywords:** Holmium, Lasers, Prostate

## Abstract

**Introduction:**

We assessed the efficacy and safety of holmium laser enucleation of the prostate (HoLEP) in patients with high comorbidity burden.

**Materials and methods:**

Data from patients treated with HoLEP at our academic referral center from March 2017 to January 2021 were prospectively collected. Patients were divided according to their CCI (Charlson Comorbidity Index). Perioperative surgical data and 3-month functional outcomes were collected.

**Results:**

Out of 305 patients included, 107 (35.1%) and 198 (64.9%) were classified as CCI ≥ 3 and < 3, respectively. The groups were comparable in terms of baseline prostate size, symptoms severity, post-void residue and Qmax. The amount of energy delivered during HoLEP (141.3 vs. 118.0 KJ, p=0.01) and lasing time (38 vs 31 minutes, p=0.01) were significantly higher in patients with CCI ≥ 3. However, median enucleation, morcellation and overall surgical time were comparable between the two groups (all p>0.05). Intraoperative complications rate (9.3% vs. 9.5%, p=0.77), median time to catheter removal and hospital stay were comparable between the two cohorts. Similarly, early (30 days) and delayed (>30 days) surgical complications rates were not significantly different between the two groups. At 3-month follow up, functional outcomes using validated questionnaires did not differ between the two groups (all p>0.05).

**Conclusions:**

HoLEP represents a safe and effective treatment option for BPH also in patients with high comorbidity burden.

## INTRODUCTION

Benign prostatic hyperplasia (BPH) is a condition characterized by an increased proliferation of both epithelial and stromal tissue, especially in the periurethral zone of the prostate (1). The prevalence of BPH substantially increases with advanced age with a reported prevalence ranging from 8% to 60% in the adult population (2). BPH can cause bothersome lower urinary tract symptoms (LUTS), including storage, voiding and post-micturition disturbances variously combined together (3, 4), ultimately impairing overall quality of life (5, 6). According to current European Association of Urology (EAU) guideline, transurethral resection of prostate (TURP) still represents the standard surgical treatment for BPH patients, unresponsive to medical therapy (3). More recently holmium laser enucleation of the prostate (HoLEP) has meaningfully revolutionized the surgical approach to LUTS/BPH, showing remarkable perioperative outcomes and long-term functional results also for larger prostate sizes (7-9), with the additional benefit of lower bleeding and blood transfusions (10).

In this scenario, patients with severe cardiovascular, metabolic and respiratory diseases typically have limited options when it comes to surgical treatment for BPH. Most importantly, such patients often take antiplatelet (AP) and/or anticoagulant (AC) medications, thus increasing the risk for postoperative bleeding and overall postoperative complications. Given these premises, HoLEP could represent a feasible and effective treatment option in this particular subset of patients due to its remarkable hemostatic properties and lower bleeding-associated complications as compared to standard TURP (11). Recent studies pointed to HoLEP being an effective treatment in elderly patients (12, 13). However, to date, only little evidence is available on the safety and efficacy of HoLEP in patients with high comorbidity burden and current limitations include limited data on short- and mid-term complications (14-17). Hence, we designed this retrospective study starting from the hypothesis that HoLEP in comorbid patients might be characterized by a non-inferior safety and efficacy profile, as compared to a cohort of matched healthy patients.

To address this unmet need, in the present study we aimed to report the safety and efficacy of HoLEP in patients with high comorbidity burden by evaluating both perioperative and functional outcomes, assessed by validated questionnaires.

## MATERIALS AND METHODS

### Patient dataset

Clinical and surgical data from patients undergoing HoLEP at our academic referral Center from March 2017 to January 2021 were prospectively collected. The study was conducted in accordance with the ethical principles of the Declaration of Helsinki and all patients signed a written informed consent before enrollment. Main inclusion criteria at baseline were: 1) symptomatic BPH not responsive to medical therapy, according to EAU guidelines (3); 2) Preoperative max flow rate (Qmax) at flowmetry < 15 mL/sec and/or post-voiding residual (PVR) > 100 mL; 3) Prostate > 60 gr. Patients with a prostate specific antigen (PSA) ≥ 4 ng/mL or suspect rectal examination underwent multiparametric magnetic resonance imaging (mpMRI) to rule out concomitant prostate cancer. Patients with persistent clinical or image-based suspect of prostate cancer were excluded from the study. Preoperative features including age, gender, body mass index (BMI) and comorbidity status assessed by Charlson Comorbidity Index (CCI), and the American Society of Anesthesiologists (ASA) physical status (PS) classification system were collected. Early and delayed postoperative complications were defined as any event occurring ≤ 30th or > 30th postoperative day, respectively, altering the normal postoperative course and/or delaying discharge. Postoperative complications were graded according to Clavien-Dindo classification.

No special protocol from a surgical standpoint was applied for patients undergoing HoLEP with AP/AC at our Institution. However, from a medical point of view, in case of suspension of coumadin, this was replaced with low molecular weight heparin (LMWH) 5 days before the procedure, while a suspension period starting from 48 hours before the procedure was generally applied for novel oral anticoagulants. The LMWH was therefore continued postoperatively before reintroducing AC therapy for a variable period of time defined by the anesthesiologists in relation to the individual risk profile. In case of AP therapy, a LMWH with prophylactic dose was routinely applied as in any other endoscopic surgery.

### Surgical technique

Enucleation was performed with the *three-lobes* or *en-bloc* with early apical release technique, as described in previous investigations (18, 19). All procedures were carried out under general anesthesia using the 120W Versapulse holmium laser machine (Lumenis, Yokneam, Israel) with a 550-µm end laser fiber (Boston Scientific, AccuMax 550 Laser Fiber). Laser energy was set at 2 J X 45 Hz, 90 W, for enucleation and 2 J X 30 Hz, 60 W, for coagulation. A 26F Storz continuous-flow resectoscope sheath was modified by inserting the 26F inner sheath, and a laser bridge was used to stabilize the fiber. A 30° down lens was preferred. The enucleated prostatic adenoma was then morcellated using a morcellator (Lumenis, Versacut). After surgery, a 22F three-way catheter was inserted and bladder irrigation was performed using saline solution. We usually removed urethral catheter on 3rd postoperative day, in case of clear urine output. All surgical procedures were performed by a single expert surgeon.

### Outcome measures and follow-up

As HOLEP relies on contemporary and wise use of both laser and accurate pulling movements, to be more accurate in quantifying the amount of energy delivered, we decided to separately count lasing time from the total of enucleation time. In particular, enucleation time was defined as the time needed to enucleate the prostatic adenoma performed by both laser energy delivery and gentle mechanic traction. The overall surgical time included also morcellation time and hemostasis time.

Assessment visits were scheduled at screening visit on day 0 and then at 1,3,6,12-months follow up after the surgical intervention. At baseline and at follow-up visits, patients were asked to write-off the following self-administered questionnaires: IPSS (international prostate symptom score), OAB-q SF (Overactive Bladder Questionnaire-Short Form), ICIQ-SF (International Consultation on Incontinence Questionnaire-Urinary Incontinence Short Form) and the IIEF-5 (international index of erectile function). The Italian versions of the IPSS (20), of the ICIQ-SF (21), of the IIEF-5 (22) and of the OAB-q SF (23) were used.

### Endpoints

Patients were divided into two groups according to CCI (< 3 and ≥ 3). The main endpoint was to appraise any difference between the two groups according to operative time, length of hospital stay, intra- and postoperative surgical complication rates. For the study purpose, we did not establish a specific postoperative haemoglobin serum level requiring blood transfusion due to the multifactorial elements involved in the decision-making process. In particular, hemoglobin serum level as well as its descend kinetics, patient’s comorbidity burden and clinical parameters all represent key drivers to establish the need for blood transfusion. Secondary endpoints were changes in Qmax, IPSS, ICIQ-SF, IIEF-5 and OAB-q SF scores.

### Statistical Analysis

Continuous variables are presented as median (IQR: interquartile range) and differences between groups were tested by Student’s independent t-test or Mann-Whitney U-test according to their normal or not-normal distribution, respectively (normality of variables’ distribution was tested by Kolmogorov-Smirnov test). Proportional data were assessed using the Chi-square test. To assess clinical differences from baseline to follow-up the median change and test for non-parametric differences were applied. All tests were two-sided. Statistical significance was set at p <0.05. Statistical analysis was performed using SPSS v. 27 (IBM SPSS Statistics for Mac, Armonk, NY, IBM Corp).

## RESULTS

Overall, 305 patients were included in the study. Baseline features of the entire cohort stratified according to CCI are reported in Table-1. In particular, 198 (64.9%) and 107 (35.1%) patients were classified as CCI < 3 and ≥ 3, respectively. Patients with CCI ≥ 3 were older (median age 73 [IQR 69–77] vs 63 [IQR 61–70]; p < 0.001), showed a significant higher use of AP/AC therapy (42.1% vs 4.9%; p < 0.001) and reported a lower median IIEF-5 score at baseline (14 [IQR 11 – 17] vs 17 [IQR 12 – 21]; p=0.02).

**Table 1:** Preoperative characteristics of patients stratified according to Charlson Comorbidity Index.

Variables	CCI ≥ 3 patients (n=107; 35.1%)	CCI < 3 patients (n=198; 64.9%)		p-value
Preoperative characteristics				
Age (years) (median, IQR)	73 (69 – 77)		63 (61 – 70)	<0.001
BMI (kg/m^2) (median, IQR)	26 (23.7 – 28.1)		26.1 (24.4 – 28.5)	0.73
ASA score (median, IQR)	2 (1-3)		2 (1-3)	0.21
AMI (n, %)	36 (33.6)		13 (6.5)	<0.001
Diabetes (n, %)	75 (70.0)		24 (12.1)	<0.001
Peripheral vascular disease (n, %)	21 (19.6)		7 (3.5)	<0.001
CVA (n; %)	26 (24.2)		6 (3.0)	<0.001
ACs/APs therapy at surgery (n, %)	36 (33.6)		17 (8.5)	<0.001
Prostate volume (mL) (median, IQR)	110 (80 – 130)		100 (75 – 130)	0.39
Creatinine serum level (mg/dL) (median, IQR)	1 (0.9-1.2)		0.9 (0.9-1.1)	0.91
HB blood level (g/dL) (median, IQR)	14.1 (13.2-15.0)		14.9 (13.7-15.3)	0.34
Q-max (mL/s) (median, IQR)	8.2 (7.0 – 10.0)		8.7 (7.3 – 10.3)	0.47
PVR volume (mL) (median, IQR)	150 (100 – 280)		130 (100 – 250)	0.11
PSA serum level (ng/mL) (median, IQR)	5.6 (2.8 – 8.7)		4.8 (2.5 – 7.3)	0.25
IPSS score (median, IQR)	24 (21 – 28)		24 (21 – 27)	0.63
IIEF-5 score (median, IQR)	14 (11 – 17)		17 (12 – 21)	0.02
OAB-q score (median, IQR)	42 (26 – 54)		39 (26 – 53)	0.76
ICIQ-sf score (median, IQR)	0 (0 – 0)		0 (0 – 0)	0.42
QoL score (median, IQR)	4 (3 – 5)		4 (4 – 5)	0.34

AC = Anticoagulants; AMI = Acute Myocardial Infarction; AP = Antiplatelets; ASA = American Society of Anesthesiologists; BMI = Body mass index; CCI = Charlson Comorbidity Index; CVA = Cerebrovascular Accident; HB = Hemoglobin; ICIQ-q = International Consultation on Incontinence Modular questionnaire; IIEF-5 = International Index of Erectile Function; IQR = Interquartile Range; IPSS = International Prostate Symptom Score; OAB-q = Overactive Bladder questionnaire; PVR = Post-voiding residual; QoL = Quality of Life

Surgical and postoperative data are reported in Table-2. Median amount of energy delivered during HoLEP (141.3 [IQR 103.2 – 162.6] vs 118.0 [IQR 100.9 – 140.3] KJ; p = 0.01) and lasing time (38 [IQR 32 – 47] vs 31 [IQR 29 – 40] minutes; p=0.01) were significantly higher in patients with CCI ≥ 3, as compared to less comorbid patients. On the contrary, median enucleation time (51 [IQR 41-60) vs 45 (IQR 38-58); p = 0.08) and overall surgical time (100 [IQR 67-120]; vs 92 [IQR 65-115]; p=0.10) were comparable between groups. No conversion to open adenomectomy or TURP were recorded in both groups. Intraoperative complications rate did not differ between the study groups (9.3% vs 9.5%; p =0.77). Similarly, median time to catheter removal (3 [IQR 3–4] vs 3 [IQR 3–3]; p=0.16) and median hospitalization time (4 [IQR 4–5] vs 4 [IQR 4–4]; p=0.35] were comparable in patients with CCI >3 and CCI <3, respectively.

**Table 2:** Surgical outcomes of patients stratified according to Charlson Comorbidity Index.

Variables		CCI ≥ 3 patients (n=107; 35.1%)	CCI < 3 patients (n=198; 64.9%)	p-value
Surgical Outcomes				
Enucleation Technique (n, %)	*Three-lobes*	38 (35.5)	89 (44.9)	0.17
	*En-bloc*	69 (64.5)	109 (55.1)	
Overall operative time (min) (median, IQR)		100 (67 - 120)	92 (65 – 115)	0.10
Enucleation time (min) (median, IQR)		51 (41 – 60)	45 (38 – 58)	0.08
Morcellation time (min) (median, IQR)		24 (16 – 35)	23 (16 – 32)	0.17
Lasing time (min) (median, IQR)		38 (32 – 47)	31 (29 – 40)	0.01
Energy delivered (kJ) (median, IQR)		141.3 (103.2 – 162.6)	118.0 (100.9 – 140.3)	0.01
Conversion to TURP (n, %)		0 (0)	0 (0)	-
Conversion to open adenomectomy (n, %)		0 (0)	0 (0)	-
**Intraoperative complication (n, %)**		10 (9.3)	19 (9.5)	0.77
Capsule perforation		7 (6.5)	13 (6.5)	
Bladder mucosal damage		3 (2.8)	7 (3.5)	

IQR = Interquartile Range

Early (30-days) surgical complications rate was comparable in the CCI ≥3 group as compared to less comorbid patients (16.7 % vs 13.1%; p=0.51). Blood transfusions were necessary in 4 (3.7%) and 6 (3.0%) patients in the CCI ≥ 3 group and CCI<3 group, respectively. A focus on baseline comorbidity features in patients requiring blood transfusion is reported in Supplementary Table-1. Similarly, late (>30-days) surgical complications were comparable between the two cohorts (1.8 % vs 1.5 %; p=0.69). As concerns management of complications, postoperative fever and orchiepididymitis were treated by antibiotics administration. In only one case of postoperative fever, it was necessary to replace vesical catheter in a patient with high comorbidity burden. There was no significant difference in the rate of postoperative bladder clot retention requiring reintervention in the CCI ≥ 3 group as compared with the counterpart (1.8% vs 1.0%, p=0.43). Acute urinary retention after discharge was managed by catheter replacement, occurring in only 2 patients in the CCI ≥ 3 group. Finally, the evidence of late postoperative urethral stricture was managed by transurethral urethrotomy under direct vision. A summary of complications and their management is reported in Table-3.

**Table 3:** Postoperative and Functional Outcomes of patients stratified according to Charlson Comorbidity Index.

Variables		CCI ≥ 3 patients (n=107; 35.1%)	CCI < 3 patients (n=198; 64.9%)	p-value
Postoperative and Functional Outcomes				
Hospitalization time (days) (median, IQR)		4 (4 - 5)	4 (4 - 4)	0.35
Catheterization time (days) (median, IQR)		3 (3 - 4)	3 (3 - 3)	0.16
Δ decreasing HB (g/dL) (median, IQR)		-0.8 (0.4 - 1.4)	-0.65 (0.4 - 1.2)	0.45
Postoperative complications (n, %)	Early events	18 (16.7)	26 (13.1)	0.51
	**CD ≤ 2**	**16 (14.9)**	**24 (12.1)**	
	Blood Transfusion	4 (3.7)	6 (3.0)	
	Fever	8 (7.4)	14 (7.0)	
	Orchiepididymitis	4 (3.7)	4 (2.2)	
	**CD >2**	**2 (1.8)**	**2 (1.0)**	
	Clot retention requiring reintervention	2 (1.8)	2 (1.0)	
	Late events	2 (1.8)	3 (1.5)	0.69
	**CD ≤ 2**	**0 (0.0)**	**1 (0.5)**	
	AUR requiring catheter replacement	0 (0.0)	1 (0.5)	
	**CD >2**	**2 (1.8)**	**2 (1.0)**	
	Urethral stricture requiring reintervention	2 (1.8)	2 (1.0)	
3-mo Q-max (mL/s) (median, IQR)		17 (14 - 21)	19 (16 – 22)	0.05
3-mo PVR volume (mL) (median, IQR)		50 (0 - 90)	40 (0 – 90)	0.68
3-mo PSA (ng/mL) (median, IQR)		0.9 (0.63 – 1.00)	0.9 (0.68 – 1.60)	0.17
3-mo IPSS (median, IQR)		8 (2 – 10)	7 (1 – 9)	0.24
3-mo IIEF-5 (median, IQR)		15 (11 – 17)	17 (12 – 21)	0.04
3-mo OAB-q (median, IQR)		15 (13 – 19)	13 (13 – 16)	0.10
3-mo ICIQ-sf (median, IQR)		0 (0 – 0)	0 (0 – 0)	0.31
3-mo QoL (median, IQR)		1 (0 – 2)	1 (0 – 1)	0.13
UI at 3-mo follow-up (n, %)		8 (7.4)	14 (7.0)	0.22
Follow-up (month) (median, IQR)		18 (9-29)	17 (9-27)	0.35

AUR: Acute Urinary Retention; CD: Clavien-Dindo; ICIQ-q: International Consultation on Incontinence Modular questionnaire; IIEF-5: International Index of Erectile Function; IQR: Interquartile Range; IPSS: International Prostate Symptom Score; OAB-q: Overactive Bladder questionnaire; PVR: Post-voiding residual; QoL: Quality of Life; UI: Urinary Incontinence; Δ: Difference between 1st postoperative day and baseline value

At 3-month follow-up, median Qmax, PSA serum level, PVR volume, as well as questionnaire scores assessing patients’ symptoms did not differ between the two groups (all p>0.05) except for IIEF-5, being lower in the more comorbid group (15 [IQR 13 – 19] vs 17 [IQR 12 – 21], p=0.04]. Urinary incontinence rate at 3 months was also comparable (7.4% vs 7.0%; p=0.22) in CCI ≥ 3 and <3, respectively (Table-3).

## DISCUSSION

While current literature contains a plethora of evidence exploring the safety of various techniques for the surgical management of BPH, there is far less investigation into the HoLEP field in the setting of high comorbidity patients. In the current paper we demonstrated that, in experienced hands, HoLEP represents a safe and effective procedure for the management of BPH also in patients with a high comorbidity burden, providing comparable perioperative and functional outcomes to those of less comorbid patients.

The first key finding of our study is that HoLEP showed outstanding early and delayed Clavien-Dindo ≤ 2 complications rate in both patient cohorts. Of note, only 4 (3.7%) patients in CCI ≥ 3 group required blood transfusions postoperatively, while only 2 (1.8%) patients experienced Clavien-Dindo>2 delayed complications. Such results are even more remarkable if we think that more than a third of patients in CCI ≥ 3 cohort continued AC/AP therapy perioperatively. The observed benefit of HoLEP in maintaining hemostasis in AC/AP patients is likely due to the physics of the holmium laser (24, 25). Indeed, due to the chromophore of water and minimal tissue depth penetration, holmium laser is able to achieve quick vaporization and coagulation of tissue without the disadvantage of deep tissue penetration (24). This characteristic of the holmium laser allows for rapid hemostasis, which is pivotal when managing patients taking AC/APs. The issue of HoLEP in AC/AP patients was first introduced by Hochreiter et al., reporting results of 19 patients on oral AC with none blood transfusion needed and only 2 patients requiring clot evacuation (26). Similarly, Tyson et al. reported perioperative results in 39 patients treated with HoLEP on either aspirin or coumadin therapy, showing a promising safety profile, since no patient received blood transfusions, although nearly 8% experienced significant postoperative hematuria and hospital readmission (27). More recently, Bishop et al. compared 52 patients on AP/AC therapy versus 73 not on therapy, reporting a transfusion rate of nearly 8% in the AP/AC group, significantly higher than the one reported in our series. (28). In this regard, in our experience median amount of energy delivered during entire procedure (141.3 vs 118.0 KJ) and lasing time (38 vs 31 minutes) were significantly higher in patients with CCI ≥ 3, as compared to less comorbid patients, probably reflecting a greater attention in hemostasis in AC/AP patients. However, a recent retrospective cohort analysis showed that AP/AC patients had a shorter overall procedure length as compared to less comorbid patients, which is in slight contrast with our findings (16). Overall, the above-mentioned differences among the studies are hardly explainable, however it should be kept in mind that HoLEP is a strongly dependent operator procedure. As such it should not be surprising that operative time may be meaningfully influenced by surgeon experience, type of fiber used, laser setting and, of course, enucleation technique employed (29).

The second key finding is that the higher amount of energy delivered in CCI ≥ 3 patients did not negatively influence health-related quality of life or functional outcomes after HoLEP. Indeed, no significant difference between the two groups were observed according to median postoperative in ICIQ-SF, OAB-q SF and QoL scores at 3 month-evaluation. As such, higher lasing time and amount of energy delivered did not necessarily translate into worse irritative symptoms in the very next period after HoLEP. We could speculate that laser setting is likely to play a key role in addressing functional outcomes but other elements are probably more critical. In particular, maintaining an anatomical dissection plane, reducing traction to the sphincter during the enucleation and avoiding capsular perforation are together crucial to allow a fast recover from irritative symptoms following HoLEP (30). Recent evidence also demonstrated the importance of the apical release in the very beginning of the procedure to maximize functional success (18, 31, 32). Moreover, efficacy in relieving BPH-related obstructive symptoms was equally satisfactory both in CCI ≥ 3 and less comorbid patients, as proved by comparable IPSS and Qmax between the two groups. This bolsters the concept that HoLEP is an effective treatment option also in case of comorbid patients since once the dissection plane is found it can be developed maintaining a bloodless surgical field in the majority of cases without compromising the fulfilling of the enucleation (33).

The main limitations of the current paper are the relatively low sample size and the short follow up, which might have introduced statistical bias. Second, this was a retrospective review of a prospectively collected database, thus the study design might have weakened itself the reliability of evidence reported. Third, all cases were performed by a single highly trained surgeon with an extensive experience in endoscopic surgery. As such, our findings could not be applicable to all surgeon- or center-related scenarios. Finally, influential conditions possibly affecting BPH-related LUTS were not evaluated, including metabolic syndrome, androgen deficiency, physical activity and smoking habits.

Despite of these limitations, the findings of the current series provide a robust foundation to assess efficacy and safety of HoLEP for the surgical management of patients with wide comorbidity burden. Further prospective, randomized, placebo-controlled studies with larger cohorts and longer follow-up will be needed to confirm the findings of the current series.

## CONCLUSIONS

Our experience confirms that in in this retrospective study with selected cases HoLEP represents a safe and effective option for the treatment of BPH also for high comorbidity patients (CCI ≥ 3). The excellent profiles of time-efficiency and the extremely low rate of clinically relevant early and delayed complications support the safety of this technique also in a real-life context within a non-preoperatively selected cohort of patients.

## Supplementary Table 1

**Table:** Baseline comorbidity features in patients requiring blood transfusion. AMI = Acute Myocardial Infarction; CVA = Cerebrovascular Accident; IQR = Interquartile Range

Variables	CCI ≥ 3 patients (n=4; 3.7%)	CCI < 3 patients (n=6; 3.0%)	p-value
Postoperative and Functional Outcomes			
Age (years) (median, IQR)	73 (69 – 77)	63 (61 – 70)	<0.001
AMI (n, %)	2 (1.8)	1 (0.5)	0.21
Diabetes (n, %)	4 (3.7)	4 (2.0)	0.23
Peripheral vascular disease (n, %)	1 (0.9)	0 (0.0)	0.60
CVA (n; %)	0 (0.0)	0 (0.0)	-

## References

[1] Roehrborn CG (2008). Pathology of benign prostatic hyperplasia. Int J Impot Res.

[2] Lim KB (2017). Epidemiology of clinical benign prostatic hyperplasia. Asian J Urol.

[3] Gratzke C, Bachmann A, Descazeaud A, Drake MJ, Madersbacher S, Mamoulakis C (2015). EAU Guidelines on the Assessment of Non-neurogenic Male Lower Urinary Tract Symptoms including Benign Prostatic Obstruction. Eur Urol.

[4] McVary KT, Roehrborn CG, Avins AL, Barry MJ, Bruskewitz RC, Donnell RF (2011). Update on AUA guideline on the management of benign prostatic hyperplasia. J Urol.

[5] Martin S, Lange K, Haren MT, Taylor AW, Wittert G, Members of the Florey Adelaide Male Ageing Study (2014). Risk factors for progression or improvement of lower urinary tract symptoms in a prospective cohort of men. J Urol.

[6] Fitzpatrick JM (2006). The natural history of benign prostatic hyperplasia. BJU Int.

[7] Zhong J, Feng Z, Peng Y, Liang H (2019). A Systematic Review and Meta-analysis of Efficacy and Safety Following Holmium Laser Enucleation of Prostate and Transurethral Resection of Prostate for Benign Prostatic Hyperplasia. Urology.

[8] Kim A, Hak AJ, Choi WS, Paick SH, Kim HG, Park H (2021). Comparison of Long-term Effect and Complications Between Holmium Laser Enucleation and Transurethral Resection of Prostate: Nations-Wide Health Insurance Study. Urology.

[9] Magistro G, Schott M, Keller P, Tamalunas A, Atzler M, Stief CG (2021). Enucleation vs. Resection: A Matched-pair Analysis of TURP, HoLEP and Bipolar TUEP in Medium-sized Prostates. Urology.

[10] Suardi N, Gallina A, Salonia A, Briganti A, Dehò F, Zanni G (2009). Holmium laser enucleation of the prostate and holmium laser ablation of the prostate: indications and outcome. Curr Opin Urol.

[11] Westhofen T, Schott M, Keller P, Tamalunas A, Stief CG, Magistro G (2021). Superiority of Holmium Laser Enucleation of the Prostate over Transurethral Resection of the Prostate in a Matched-Pair Analysis of Bleeding Complications Under Various Antithrombotic Regimens. J Endourol.

[12] Yilmaz M, Esser J, Suarez-Ibarrola R, Gratzke C, Miernik A (2022). Safety and Efficacy of Laser Enucleation of the Prostate in Elderly Patients - A Narrative Review. Clin Interv Aging.

[13] Agarwal DK, Large T, Stoughton CL, Heiman JM, Nottingham CU, Rivera ME (2021). Real-World Experience of Holmium Laser Enucleation of the Prostate with Patients on Anticoagulation Therapy. J Endourol.

[14] El Tayeb MM, Jacob JM, Bhojani N, Bammerlin E, Lingeman JE (2016). Holmium Laser Enucleation of the Prostate in Patients Requiring Anticoagulation. J Endourol.

[15] Goldman L (1983). Cardiac risks and complications of noncardiac surgery. Ann Intern Med.

[16] Rivera M, Krambeck A, Lingeman J (2017). Holmium Laser Enucleation of the Prostate in Patients Requiring Anticoagulation. Curr Urol Rep.

[17] Sun J, Shi A, Tong Z, Xue W (2018). Safety and feasibility study of holmium laser enucleation of the prostate (HOLEP) on patients receiving dual antiplatelet therapy (DAPT). World J Urol.

[18] Tuccio A, Grosso AA, Sessa F, Salvi M, Tellini R, Cocci A, a t (2021). l. En-Bloc Holmium Laser Enucleation of the Prostate with Early Apical Release: Are We Ready for a New Paradigm?. J Endourol.

[19] Grosso AA, Di Maida F, Mari A, Nardoni S, Tuccio A, Minervini A (2022). Holmium laser ablation of the prostate (HoLAP) with moses technology for the surgical treatment of benign prostatic hyperplasia. Int Braz J Urol.

[20] Russo F, Di Pasquale B, Romano G, Vicentini C, Manieri C, Tubaro A (1998). International prostate symptom score: medico e paziente a confronto. Arch Ital Urol Androl.

[21] Tubaro A, Zattoni F, Prezioso D, Scarpa RM, Pesce F, Rizzi CA (2006). Italian validation of the International Consultation on Incontinence Questionnaires. BJU Int.

[22] D’Elia C, Cerruto MA, Cavicchioli FM, Cardarelli S, Molinari A, Artibani W (2012). Critical points in understanding the Italian version of the IIEF 5 questionnaire. Arch Ital Urol Androl.

[23] McKown S, Abraham L, Coyne K, Gawlicki M, Piault E, Vats V (2010). Linguistic validation of the N-QOL (ICIQ), OAB-q (ICIQ), PPBC, OAB-S and ICIQ-MLUTSsex questionnaires in 16 languages. Int J Clin Pract.

[24] Gravas S, Bachmann A, Reich O, Roehrborn CG, Gilling PJ, De La Rosette J (2011). Critical review of lasers in benign prostatic hyperplasia (BPH). BJU Int.

[25] Habib EI, ElSheemy MS, Hossam A, Morsy S, Hussein HA, Abdelaziz AY (2021). Holmium Laser Enucleation Versus Bipolar Plasmakinetic Resection for Management of Lower Urinary Tract Symptoms in Patients with Large-Volume Benign Prostatic Hyperplasia: Randomized-Controlled Trial. J Endourol.

[26] Hochreiter WW, Thalmann GN, Burkhard FC, Studer UE (2002). Holmium laser enucleation of the prostate combined with electrocautery resection: the mushroom technique. J Urol.

[27] Tyson MD, Lerner LB (2009). Safety of holmium laser enucleation of the prostate in anticoagulated patients. J Endourol.

[28] Bishop CV, Liddell H, Ischia J, Paul E, Appu S, Frydenberg M (2013). Holmium Laser Enucleation of the Prostate: Comparison of Immediate Postoperative Outcomes in Patients with and without Antithrombotic Therapy. Curr Urol.

[29] Tuccio A, Sessa F, Campi R, Grosso AA, Viola L, Muto G (2020). En-bloc endoscopic enucleation of the prostate: a systematic review of the literature. Minerva Urol Nefrol.

[30] Porreca A, Colicchia M, Tafuri A, D’Agostino D, Busetto GM, Crestani A (2022). Perioperative Outcomes of Holmium Laser Enucleation of the Prostate: A Systematic Review. Urol Int.

[31] Tuccio A, Grosso AA, Di Maida F, Mari A, Minervini A (2022). Letter to the Editor regarding the article “The “Omega Sign”: a novel HoLEP technique that improves continence outcomes after enucleation”. World J Urol.

[32] Tunc L, Yalcin S, Kaya E, Gazel E, Yılmaz S, Aybal HC (2021). The “Omega Sign”: a novel HoLEP technique that improves continence outcomes after enucleation. World J Urol.

[33] Rücker F, Lehrich K, Böhme A, Zacharias M, Ahyai SA, Hansen J (2021). A call for HoLEP: en-bloc vs. two-lobe vs. three-lobe. World J Urol.

